# EXPERIENCE OF OLDER ADULTS WITH OBJECTIVELY MEASURED PHYSICAL ACTIVITY IN FALL PREVENTION INTERVENTION

**DOI:** 10.1093/geroni/igae098.2713

**Published:** 2024-12-31

**Authors:** Eunice Ojo, Victoria Loerzel, Ladda Thiamwong

**Affiliations:** University of Central Florida Orlando Florida, Orlando, Florida, United States; University of Central Florida, Orlando, Florida, United States; University of Central Florida, Orlando, Florida, United States

## Abstract

Falls are a preventable event that negatively affects the health of older adults. Unfortunately, they have many risks for falls including impaired gait and balance, and muscle weakness. Objective assessment of older adults’ physical activity with portable technology including accelerometers helps plan fall prevention interventions but there is limited information about their experience. This study aimed to explore participants’ experiences with physical activity measured with an accelerometer. A qualitative descriptive study was conducted among 23 older adults, 60 years, and older living in low-income community settings in Central Florida. Participants were included if they participated in the Physio-Feedback Exercise Program (PEER) study that involved using a wrist-worn accelerometer (Fitbit) to measure physical activity. A semi-structured interview guide was used to obtain participants’ experiences with physical activity and the technology used. Participants were individually interviewed, and audio recorded. They were offered an incentive of $20 for participating in the qualitative study. The responses were transcribed verbatim, coded, and analyzed using thematic analysis. Two major themes and sub-themes emerged from the data. These included 1) Barriers to physical activity (physical limitation, challenges with technology, fear of falling), and facilitators of physical activity (feedback from the accelerometer, progress monitoring, self-motivation). The use of the accelerometer encouraged participants’ engagement in physical activity. Reducing barriers and encouraging facilitators of physical activity with accelerometers could promote fall prevention interventions. Future studies should include exploring the experience of cognitively impaired older adults’ engagement in physical activity with the accelerometer.

